# Connecting the dots: sex, depression, and musculoskeletal health

**DOI:** 10.1172/JCI180072

**Published:** 2024-09-17

**Authors:** Mackenzie Newman, Henry J. Donahue, Gretchen N. Neigh

**Affiliations:** 1 Department of Orthopaedic Surgery, Virginia Commonwealth University School of Medicine, Richmond, Virginia, USA.; 2 Department of Biomedical Engineering, Virginia Commonwealth University College of Engineering, Richmond, Virginia, USA.; 3 Department of Anatomy and Neurobiology, Virginia Commonwealth University School of Medicine, Richmond, Virginia, USA.

## Abstract

Depression and multiple musculoskeletal disorders are overrepresented in women compared with men. Given that depression is a modifiable risk factor and improvement of depressive symptoms increases positive outcomes following orthopedic intervention, efforts to improve clinical recognition of depressive symptoms and increased action toward ameliorating depressive symptoms among orthopedic patients are positioned to reduce complications and positively affect patient-reported outcomes. Although psychosocial factors play a role in the manifestation and remittance of depression, it is also well appreciated that primary biochemical changes are capable of causing and perpetuating depression. Unique insight for novel treatments of depression may be facilitated by query of the bidirectional relationship between musculoskeletal health and depression. This Review aims to synthesize the diverse literature on sex, depression, and orthopedics and emphasize the potential for common underlying biological substrates. Given the overrepresentation of depression and musculoskeletal disorders among women, increased emphasis on the biological drivers of the co-occurrence of these disorders is positioned to improve women’s health.

## Parallels in sex differences: depression and musculoskeletal health

Sex differences in both depression (1, 2) and musculoskeletal health (3, 4) are well established. Depression and related disorders affect 4 of 10 people across the globe, and the incidence is overrepresented 2:1 in women compared with men (5). Moreover, sex differences in depression are consistently reported beginning in adolescence and extending across the lifespan (1). Similar to the overrepresentation of depression in women, multiple musculoskeletal ailments are also overrepresented in women (6). In young women, damage to the anterior cruciate ligament (ACL) is up to 5 times more common than in young men (6, 7). In the context of aging, osteoporosis, a pathological reduction in bone mineral density (BMD) and deterioration of bone microarchitecture, is also overrepresented in women as compared with men (8). Differences in levels of physical activity by sex throughout the lifespan, with men typically reporting greater degrees of daily physical activity than women (9, 10), may contribute to sex differences in both depression and musculoskeletal health, but this explanation is incomplete. For instance, although physical activity was a key contributor to recovery from hip fracture for men, physical activity did not associate with metrics of resilience following hip fracture in women (11). For the purposes of this Review, we focus on sex as a biological factor. Gender is an important variable in both the understanding of depression (12) and musculoskeletal health (13, 14) and, with continued attention to carefully delineating biological sex from gender in research designs, data will become available to fill the current gaps in understanding regarding the variable of gender, as an intersecting factor with biological sex, on both brain and musculoskeletal health (14).

**Figure 1:**
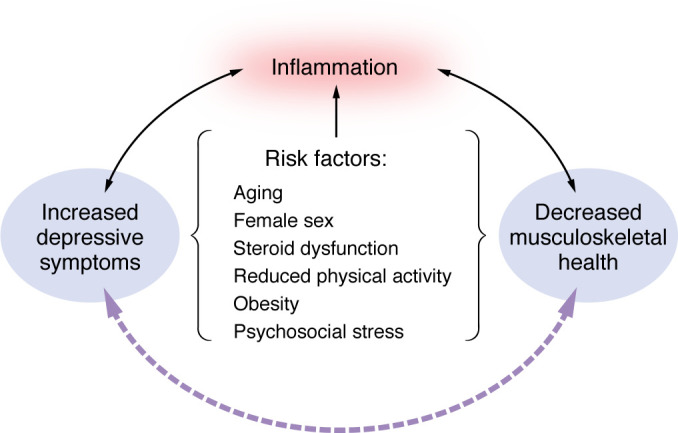
Relationships among risk factors common to depression and decreased musculoskeletal health. The schematic highlights inflammation as the common link between these conditions. The solid lines represent established relationships, and the dashed line represents the proposed relationship explored in this Review.

Attention to underlying mechanisms that fuel the correlation between depression and musculoskeletal health may generate novel therapeutic avenues that can improve overall health. To this end, a recent analysis of the National Health and Nutrition Examination Survey database demonstrated that osteoarthritis (OA), a musculoskeletal disorder that affects both cartilage and bone, was positively associated with depression, and depression mediated the association between OA and cardiovascular mortality (15). Inflammation is a known underlying factor common to depression and OA as well as a mediator for increased mortality risk when these conditions are comorbid (16). In this Review, we highlight known associations among depression and musculoskeletal conditions, including inflammation, emphasizing implications and opportunities for women’s health (Figure 1). Our discussion will focus on cisgender women and cisgender men, as these are the groups primarily represented in the available literature.

The existence of a bone-to-brain axis first began to be recognized in the 1970s, with observations of the paradoxical effect of traumatic brain injury on fracture healing (17, 18), and study of the bone-to-brain axis largely focused on the relationships between bone health and neurodegeneration (19, 20). It is well established that aging leads to cognitive decline as well as osteopenia and sarcopenia, conditions of low bone and skeletal muscle mass, respectively. More recently, appreciation for interactions along the bone-to-brain axis have begun to consider mental health. Over the past 20 years, there has been an 80-fold increase in publications related to mental health and orthopedics, with a doubling from 2019 to 2021 (21). Significant depression symptoms have been reported among 35%–60% of patients waiting for orthopedic interventions, and many of these patients are not yet in care for depression (22, 23). Among those awaiting orthopedic interventions who report depressive symptoms, women are overrepresented, consistent with the extant literature on depression (22, 23). Importantly, while directionality is inherently difficult to discern in human studies, intervention studies consistently demonstrate that improving symptoms of depression can enhance orthopedic outcomes, including reducing pain and improving function (24, 25); however, the reverse is not necessarily true such that reducing pain and improving function is not clearly associated with improvements in depressive symptoms (26). The directionality of these observations demonstrates that depressed mood is not always simply a psychological response to change in physical ability or consequence of pain and can be a distinct biological event that requires primary attention (26). To this end, understanding the bidirectional relationship between depression and musculoskeletal health, and how biological sex may modify this relationship, is an essential step in precision medicine.

## Exercise: early insight into depression and musculoskeletal health

Physical inactivity is detrimental to both the brain and musculoskeletal system, and an emerging concept is that disuse and physical inactivity induces physiological changes similar to those seen with aging (27, 28). Conversely, exercise has been proposed as a panacea to prevent and treat common disorders and diseases (29), and the positive effects of exercise extend to depression, highlighting the physical nature of depression (30). For instance, a prospective study of a healthy cohort of over 33,000 adults in Norway reported that 12% of depression cases could be prevented through engagement in at least 1 hour of physical activity per week (31). In terms of treatment, the first published study documenting exercise as an effective therapeutic intervention for women with depression was published over 40 years ago (32). The first systematic study in men and women followed nearly 20 years later, and it showed that exercise was as effective as pharmacological antidepressant therapy in reducing depressive symptoms in both sexes (33). At present, thousands of studies have consistently demonstrated that exercise is a positive intervention for depression. A recent systematic review of 218 studies found that exercise was consistently associated with moderate reductions in depression, with greater effect sizes in the investigations that included more women among the participants (34). While the social aspects that can accompany exercise may also be important in the prevention and amelioration of depression (35), exercise interventions appear to be the most impactful for depression when physical benefits are also present, suggesting that the influence extends beyond the social level. For instance, the positive effects of exercise on depression are more robust among frail older individuals (36), and exercise is more impactful on symptoms of depression when benefits on bone health are also observed (37), suggesting that the greatest effects of exercise on depression are produced when there is a positive effect on musculoskeletal health. Collectively, the demonstration of a positive effect of exercise on both mental and physical health are compelling for both men and women with potential for added value in women.

The multifaceted effects of exercise may be supported by effects of exercise on muscle, bone, and adipose, which are highly plastic tissues. Generally, remodeling of one of these compartments engages remodeling of the others, and all three have robust signaling cascades capable of endocrine communication. For instance, with aging, there can be as much as a 50% loss of muscle strength and a 30%–50% loss of bone density, which frequently co-occur with an increase in adipose tissue (38). Beyond the immediate effects of increases in muscle and bone and decreases in adiposity, studies on the positive effects of exercise point to increased suppression of low-grade inflammation as a key mechanism, particularly in postmenopausal women (39, 40) and elderly men (41). Emphasizing the widely appreciated positive effects of exercise, there is growing interest in identifying exercise mimetics to generate the pleiotropic effects of exercise in the absence of increased physical activity (42). Developing an understanding of the mechanisms that underlie the biological relationships among exercise, depression, and musculoskeletal health in women and men may provide additional avenues of intervention for those in which exercise is not feasible or sufficiently efficacious.

## Comorbidity of depression and musculoskeletal conditions

Depression has been reported in multiple musculoskeletal conditions, including a strong representation among conditions with a chronic inflammatory component such as OA and rheumatoid arthritis (RA) (43). In the case of RA, inflammatory mechanisms are a prominent area of study for the comorbidity of RA and depression, and preclinical evidence suggests that blockage of neuroinflammation may alleviate depressive symptoms in a rodent model of RA (44). Disentangling mechanisms underlying comorbidity of depression with RA and OA is challenging due to the chronic nature of the conditions and the reverberating cycles that can be created among pain, inflammation, activity, and physical function, which all impact mood (45). For instance, individuals diagnosed with both obesity and depression are the most likely to require total knee arthroplasty within 5 years of diagnosis of knee OA (46). However, depressive symptoms only associate with higher reporting of pain in individuals with a BMI ≤25, suggesting that body mass alone cannot account for this relationship (47). Multiple additional factors may influence the relationship between depression and OA, including activity level (48) and inflammation (16). Furthermore, patients with comorbid depression and OA are less responsive to available pharmacological therapies (43). While the clinical importance of recognizing depression in the context of OA and RA is critical, disentangling mechanisms of comorbidity, beyond inflammation, is hampered by the chronicity of progressive nature of both OA and RA.

While depression appears to follow OA and RA, manifestation of depression has been proposed to precede, as well as follow, osteoporosis (49, 50), and the relationship does not appear to be as consistently mediated by underlying inflammation (51). An association between osteoporosis and depression has been long recognized (49, 52–54). Research into osteopenia, the hallmark precursor to osteoporosis, has shown that decreases in BMD are enriched in elderly populations, particularly in women with depression (53, 55, 56), putting them at higher risk for spine and hip fractures (53, 56–58). Meta-analysis of 14 studies related to BMD and depression in women found that overall depression was associated with a significant decrease in mean BMD in spine and hip compared with women without significant depressive symptoms (56). Importantly, the relationship between BMD and depression is not fully explained by side effects of antidepressant use or other common confounders (57). Furthermore, psychotropic medications do not independently account for the relationship between incident fractures and depression history (58). Interestingly, genetic association between depression and fracture has recently been reported that suggests common genetic architecture may contribute to risk for both depression and compromised bone integrity (59), and a Mendelian randomization analysis demonstrated a causal link between genetically predicted depression and risk of osteoporosis in perimenopausal women (60). In addition, the well-established roles of steroids, both glucocorticoids and sex steroids (estrogens and androgens), in the biology of depression and musculoskeletal health (53, 61, 62) are key areas of continued study with a goal of developing interventions that do not produce the risky side effect profiles of blunt steroid-based interventions (42).

## Depression is a modifiable risk factor for orthopedic complications

Depression is common among individuals seeking orthopedic surgical intervention and is overrepresented in these individuals compared with the general population (22, 23). This may be in part because depression has been identified as a risk factor for injury that may require surgical intervention, particularly in elderly populations (56, 58, 63). Collectively, the impact of preoperatively diagnosed psychiatric comorbidities is associated with significantly higher postoperative care costs across all orthopedic procedures (64). The substantial risk of unremitted depression for patients undergoing orthopedic surgical intervention has been noted as a risk factor for infection after total joint arthroplasty on par with bacterial colonization, cardiovascular, and renal diseases, obesity, diabetes, anemia, malnutrition, tobacco use, and alcohol consumption (65). In addition, preoperative depression is associated with prolonged opioid use after total knee arthroplasty (66) or shoulder arthroplasty (67). Systematic review of orthopedic surgical outcomes and preexisting mental health diagnoses in those under age 65 demonstrated that, in 83% of the studies considered, individuals with preexisting mental health diagnoses had diminished postoperative outcomes, including higher pain and worse functional outcomes. Furthermore, individuals with depression were the most likely to need additional postoperative interventions (68). Similar results have been reported following distal radius fracture surgery, with patients with preoperative depression diagnoses exhibiting increased complications, including infection, emergency department visits for pain, and hardware complications (69).

In addition to readmissions for infection or revision, outcomes following surgical orthopedic intervention are also commonly assessed by patient-reported outcomes (PROMs). PROMs are self-reported survey metrics from patients — including function, pain, and emotional well-being — that can be assessed both before and after treatment intervention to capture the patient perspective on the value of an intervention and associated quality of life (70). Patient-reported low emotional well-being scores or depression before surgery have been found to be associated with poorer surgical outcomes, largely in pain management and overall satisfaction (64, 68, 69, 71–80). While significant sex differences were not reported in all studies (73, 74, 78–80), in some, women were noted as reporting higher prevalence of baseline depression and worse postintervention outcomes compared with men (64, 69, 71, 72, 75). Importantly, sex differences in reporting for PROMs complicate the sex differences observed from these metrics. For instance, the difference in PROMs reported by men versus women in the preoperative period exceeded the minimal clinically important difference for a group of patients undergoing total shoulder arthroplasty such that women report worse preintervention metrics than men. This baseline sex difference in PROMs could affect clinical understanding of surgical outcomes (76) and has been demonstrated in hip and knee arthroplasty (81). However, even in studies where sex differences in PROMs are evident, an impact of depression is also evident. Regardless of sex, for patients with comorbid depression and anxiety, improvement in PROM score is negatively impacted by comorbid mental health conditions (71).

While depression is linked with adverse outcomes from orthopedic intervention, it is critical to emphasize that this is a modifiable risk factor and should not be an exclusion criteria for orthopedic intervention. A recent retrospective chart review that focused on more stringent control of confounding variables, including substance use, did not find an association between mental health diagnoses and surgical outcomes after total joint arthroplasty (82), suggesting that preoperative medication optimization is an important point of focus. In addition, a systematic review of 10 studies including 33,501 patients indicated that in the majority of studies assessed, patients in active treatment for depression (pharmacological or cognitive behavioral therapy) reported lower revision of joint arthroplasty and/or improved postoperative functional outcomes (83). Recognizing and treating depression in patients seeking orthopedic care is positioned to improve both orthopedic and neuropsychiatric outcomes and may be particularly positively impactful for women given the overrepresentation of both depression and musculoskeletal disorders among women.

## Orthopedic surgery as a risk factor for depression

A small but growing literature base has begun to emerge concerning orthopedic surgical intervention as a risk factor for onset of depression. A retrospective review of insurance claims data in the United States examined first reports of the International Classification of Diseases, Ninth Revision (ICD-9) codes to identify codes consistent with depression or anxiety subsequent to ACL reconstruction. Of the 82,962 patient records reviewed, 10.7% had a new postoperative depression or anxiety diagnosis. 60.7% of female patients and 39.3% of male patients in this analysis had evidence of a new depression/anxiety diagnosis after ACL reconstruction (84). ACL reconstruction includes a substantial recovery period to return to preinjury levels of activity, and psychological barriers can influence recovery (85), including fear avoidance (86), which can prolong recovery and perpetuate pain. Interestingly, impairments in recovery from ACL reconstruction have also been proposed to be linked to learned helplessness (87), and psychological interventions aimed at targeting neural circuitry integral to fear behaviors and depression have been shown to be efficacious (88). Importantly, neuropsychiatric studies of the neurobiology of fear behaviors and learned helplessness, outside of the context of orthopedic injury, have consistently implicated neuroinflammatory signaling (89–92) and sex steroids (93–96) in pathological manifestation of fear behaviors and learned helplessness. Collectively, these data suggest that further study into the neurobiology of full return to activity following ACL reconstruction may provide additional avenues to support recovery in individuals with a delayed return to full preinjury physical activity. This may be particularly important because failure to return to the preinjury level of activity after surgery has been associated with overall reduced quality of life and health long term after injury (97).

In addition to the consideration of depression following ACL reconstruction, several longitudinal studies have examined the onset of depression following orthopedic surgery and report a sex difference similar to that observed in depression generally. A small longitudinal study of 56 patients hospitalized in the United Kingdom for either hip or knee arthroplasty tracked symptoms of depression and anxiety for the duration of the postoperative hospitalization with the Hospital Anxiety and Depression Scale. Over the course of hospitalization, 50% of patients developed depression, and female patients were more likely than male patients to develop significant depressive symptoms (odds ratio [OR] = 3.48) (98). In addition, postoperative depression was assessed in all orthopedic surgery patients in a multicenter cross-sectional study of 443 adult patients in Ethiopia. The study used the Patient Health Quality-9 scale with assessments conducted pre-operatively and on the day of discharge after surgery. Scores consistent with significant depressive symptoms were reported in 61.8% of patients at discharge, and female patients were more likely than male patients to report significant depressive symptoms (OR = 2.69) (99). The timing of the onset of these depressive symptoms, close to the time of the physical challenge of surgery, is consistent with the sickness behavior theory of depression (100), and one case report exists to date positing a similar manifestation of sickness behavior in humans following fracture (101).

Similar to traditional sickness responses to pathogens, inflammatory factors including chemokines and cytokines, are integral to a normal postoperative response. When the inflammatory response is prolonged or excessive, adverse consequences can manifest, and a role of inflammation in the manifestation of depression is well established outside the context of orthopedic insult (102). In addition, inflammation has been proposed to mediate the association between mood disorders and musculoskeletal disease (103). One clinical study has investigated this association in the context of orthopedic surgery: blood samples were assessed preoperatively and on the first postsurgical day for C-reactive protein (CRP) and IL-6 as markers of inflammation. Depressive symptoms were assessed with the Center for Epidemiological Studies-Depression questionnaire 2–3 weeks prior to surgery and 1 month and 3 months following surgery. Of the 110 patients that completed the study (35 male patients and 75 female patients), 51.96% of patients reported an increase in depressive symptoms at 1 month which decreased to 31.7% at 3 months following surgery. No sex difference was observed at either time point. Depressive symptoms at 1 month and 3 months were predicted by postoperative levels of CRP (indirect relationship) and IL-6 (direct relationship). No predictive relationship was observed for presurgical CRP or IL-6 on depressive symptoms, suggesting that the response to surgical trauma, and not general inflammatory state, underlies the predictive value of these biomarkers (104). Given the robust evidence for critical mechanistic effects of inflammatory factors on the brain (105–107), within and outside the context of depression, the pivotal role of inflammatory factors in the maintenance of bone and muscle (108), the profound differences in inflammatory signaling between male and female organisms (2, 105, 106), and the proinflammatory effects of early life trauma for women later in life (109), further study in the area of postsurgical depression may lead to improved patient outcomes from orthopedic as well as other types of surgical intervention.

## Additional mechanistic links between depression and musculoskeletal health

Studies focused on the implications of a bone-to-brain axis have primarily been performed in the context of neurodegenerative conditions (20). Evidence suggests that neuronal factors and hormones affect bone and bone-derived factors affect the brain (110). For instance, oxytocin and vasopressin, hormones of the neurohypophyseal system, are anabolic and catabolic, respectively, to bone (111, 112). On the other hand, osteocalcin, a hormone secreted from bone, affects hippocampal development and cognitive function (110, 113). Additionally, there exists a bone-skeletal muscle axis. For instance, skeletal muscle–derived IGF-1 and myostatin are anabolic and catabolic, respectively, to bone (114, 115) whereas bone-derived osteocalcin and sclerostin are anabolic and catabolic, respectively, to skeletal muscle (116, 117). Finally, emerging evidence suggests that skeletal muscle derived myokines effect brain function, and disruption of this signaling leads to cognitive decline (118). In this Review, we have highlighted studies that suggest that the bone-to-brain axis may also influence depression. Glucocorticoids and sex steroids, in particular estrogen, are positioned to be mechanistic contributors to a relationship between depression and musculoskeletal health, and these have recently been thoroughly detailed in several impactful reviews (45, 119, 120). For instance, there is a robust body of literature on the impact of estrogen depletion on both bone health and depression, as well as other somatic conditions (121–123). Inflammation is another key area of study that has a robust presence in both the current understanding of depression (124–127) and musculoskeletal health (128, 129). The effect of inflammation on bone health and the potential driving role of inflammatory signaling in the manifestation of depression has been emphasized in the literature (130, 131) and throughout this Review. Although we concur with the importance of continued study of both steroids and inflammation for understanding and ultimately treating depression and musculoskeletal disorders, we highlight here additional, and likely collaborative, mechanistic links between musculoskeletal health and depression. We draw attention to these mechanistic links, often overlapping with both steroids and inflammatory processes, to provoke thought and inquiry to the potential for targeted mechanistic interventions that go beyond broad-spectrum interventions on steroids or inflammatory factors that often lead to secondary problems due to the ubiquitous and pleiotropic functions of both endocrine and immune signaling.

## Myokines

As noted, bone health and muscle health are closely interlinked. We have focused primarily on bone health in this Review owing to the better-developed literature extending from the larger body of work on the bone-to-brain axis (19, 20), but the importance of muscle, as well as adipose, for the biology of sex differences in depression should not be overlooked. Sarcopenia, defined as loss of muscle mass, has widely been shown to occur at high rates with depression (132–137). Causal associations between depression and grip strength, the primary measure for sarcopenia, have been validated via Mendelian randomization; however, loss of muscle mass was not found to be associated with depression (138). While depression is more likely to have an effect on sarcopenia than osteopenia (138, 139), little data are available. Like osteopenia, a steroidal or sex-related factor is still likely to contribute (140). The steroidogenic theory of sarcopenia has been linked to changes in insulin-like growth factor-1 (IGF-1), a principal anabolic myokine (141), and development of depression has been linked to both low and high, but not median, circulating IGF-1 levels in elderly populations (142). In men, high levels of circulating IGF-1 have been linked to depression; in women, IGF-1 is inversely linked to depressive disorders (143, 144). In a genome-wide twin study looking at novel biomarkers shared between development of depression and decreases in grip strength, researchers identified nine SNPs associated with both outcomes, revealing links to androgen activity, potassium channels, rho GTPase activity, fibroblast growth factor signaling, and general cytokine pathways (132). While these findings were not sex specific, the roles of these pleiotropic loci in this context have not been explored in model systems. Although less established than links between bone health and depression in women, investigation into the mechanistic relationship between muscle health and depression in women is a critical area of further study.

## Monoamines

Early life stress and chronic stress have been associated with a myriad of musculoskeletal deficits, including reductions in bone growth (145), impaired fracture healing (146), and increased bone loss (147). In addition, early life stress is well established as a risk factor for the manifestation of depression (148). Early-life psychosocial trauma has been shown to exert sustained effects on both the hypothalamic-pituitary-adrenal (HPA) axis and its principal effectors, glucocorticoids (149) as well as on the autonomic nervous system and monoamine transmission (150). In addition to the well-established negative effects of glucocorticoids on bone (151) and in the manifestation of depression (152), alteration in monoaminergic transmission is instrumental in both bone health (19) and depression (153). Although studies to date have been conducted predominantly in male individuals, bone-related monoaminergic transmission may be an opportunity for intervention. A study of 20 patients undergoing surgery for an upper ankle fracture demonstrated that the expression of tyrosine hydroxylase, a rate-limiting enzyme in catecholamine synthesis, in the fracture hematoma correlated with patient-reported symptoms of depression, perceived stress, and pain shortly after surgery as well as with impaired healing following surgery. A follow-up study in a mouse model supported the direction of the findings and demonstrated that catecholamines and β-adrenoceptor signaling in chondrocytes mediated the detrimental effects of stress on bone health in male rodents (154). In addition, a preclinical study demonstrated that, in male rodents, administration of the beta blocker propranolol prior to an experimentally induced bone fracture prevented the negative effects of a history of chronic psychosocial stress on healing (146). Expanding the assessments of catecholamines and bone-related outcomes to include female individuals will be an important future area of study to determine the extent to which these mechanisms may be at play.

In addition to catecholamine influences, the indolamine serotonin is well established as influential in depression and in bone health. In fact, a recent review investigating the links between RA and depression highlighted the potential role of serotonin (155). Pharmacological treatments for depression frequently focus on modification of serotonergic transmission, with selective serotonin reuptake inhibitors (SSRIs) as the leading class of medications (156). One large retrospective study regarding total knee or hip arthroplasties (*N* = 20,112) found that treatment with preoperative SSRIs, but not other antidepressant drugs, was associated with a significant reduction in risk of revision surgery (157). Despite SSRI compounds having promiscuous binding affinities and efficacies (158), this study suggests a common, established mechanism shared by orthopedic surgery. As discussed earlier in this Review, long-term SSRI use has been associated with orthopedic comorbidities, such as hip or spine fracture and low BMD in a compound-specific manner (159), which causes some concern around use of SSRIs and bone health. Further investigation into the mechanisms by which SSRIs negatively affect bone health may yield more viable treatment strategies. For instance, a study in male rats demonstrated that while the SSRI escitalopram increased bone resorption and decreased bone formation, cotreatment with carbidopa, an inhibitor of peripheral serotonin synthesis, prevented the negative effects of escitalopram on bone. This suggests that the negative effects of SSRIs may be mediated by the gut, which could allow for novel therapeutic strategies to restrict SSRI effects to the brain (25). Additional investigation into the potential role of monoamines in musculoskeletal health and recovery may provide new insight into the pathophysiology of depression and identify novel treatment approaches for both disorders.

## Osteopontin and ketamine

Osteopontin (OPN), a secreted phosphoprotein, has numerous functions across organ systems, including bone, the immune system, and the nervous system (160). In addition to critical homeostatic functions, OPN has been linked with bone-related diseases, including osteoporosis (161), and plasma OPN is elevated preoperatively for patients undergoing either hip or knee arthroplasty compared with a normal reference group (162). OPN has gained attention in the context of the neurobiology of depression in part due to the presence of OPN receptors on microglial cells; activation of these receptors can lead to either NF-κB activation and thereby expression of proinflammatory cytokines or integrin-related pathways generating actin remodeling and cell migration or phagocytosis (163).

Several recent studies have considered the potential for OPN to mediate the effects of the novel, nonclassical antidepressant ketamine, which primarily acts on the glutamatergic system rather than serotonin. A study group comprising 28 medication-free people with treatment-refractory major depressive disorder and 16 healthy controls was given a single infusion of ketamine, assessed prior to infusion, and then assessed for 3 days thereafter. OPN was reduced in patients with depression compared with the control group, and ketamine increased plasma OPN in patients with depression only, with no effect on any bone markers in healthy controls (164). Conversely, assessment of markers of BMD in humans in treatment for depression following 6 infusions of ketamine demonstrated a sustained decrease in OPN in both male and female patients lasting at least 26 days after the last infusion, with demonstration of sex differences in other bone markers assessed (165). Given the female prevalence of depression, use of ketamine as a therapeutic among women is a rapidly growing area of study (166). Ketamine has been successfully used to prevent postsurgical depression in the context of Cesarean delivery (167, 168), and completion of a recent feasibility randomized trial to assess the efficacy of ketamine to prevent postsurgical depression in both men and women showed promise for further investigation (169). Given the potential positive effects of ketamine on both bone and prevention of depression, assessment of ketamine in the context of orthopedic surgery may be of value for preventing depression following orthopedic intervention and or remitting depression prior to orthopedic surgery.

## Sclerostin

As mentioned above, chronic administration of escitalopram in male rats leads to reduced bone formation and enhanced bone resorption. Of additional note in this study was the observation that deficits in bone were concomitant with elevated sclerostin (SOST) (25), and recent evidence suggests a role of SOST in the brain. SOST is a signaling factor released by osteocytes and is a primary antagonist of Wnt signaling (170). SOST is also critical in the bone morphogenetic protein (BMP) signaling pathway. Both Wnt signaling and BMP have been implicated in preclinical studies of depressive-like behaviors and neuroplasticity (171–173). For instance, acute local elevations of SOST in the brain have been shown to negatively effect social and affective-like behavior concomitant with reduced dendritic complexity in the hippocampus (174). In addition, in a rat model, estrogen depletion via chronic letrozole treatment increased SOST in the hippocampus, which was associated with impaired performance in learning and memory. Given the known role of SOST as an inhibitor of the Wnt signaling pathway, the observed effects were attributed to inhibition of this pathway (175). Recently, new data have emerged that demonstrate the ability of SOST to cross the blood-brain barrier and impair synaptic plasticity and memory in mice through inhibition of Wnt/β-catenin signaling and increased amyloid β (176).

Evidence for a potential neural role of SOST is also suggested by a few available human studies. Plasma SOST levels were elevated in older adults (177), which is perhaps not surprising given the role of SOST in bone loss and the association between osteoporosis and Alzheimer’s disease (178). Furthermore, elevated serum SOST correlates with higher cognitive impairment and bone dysmetabolism in older people (176). As further support for the role of SOST in neural function emerges, SOST may be a particularly accessible target for consideration of prevention and treatment of depression and cognitive impairment. Monoclonal antibodies against SOST are now utilized as treatment for osteoporosis (179) and may be particularly advantageous interventions for women who are disproportionately impacted by both osteoporosis and depression. Future studies that examine the incidence of depression and cognitive aging among patients treated with monoclonal antibodies to SOST for osteoporosis will provide innovative insight into these potential relationships.

## Conclusion

Sex differences in both the manifestation of depression and musculoskeletal disorders are well established, and great opportunity exists for leveraging the understanding of each group of disorders to build a gestalt understanding of multisystem interactions that could yield advances in personalized medicine. In addition to the longer term potential for development of novel treatment interventions, patient lives could be immediately positively impacted through more comprehensive approaches to orthopedic care with an emphasis on assessing and treating symptoms of depression. Increased recognition among orthopedic care providers of the physical effects of depression, and appreciation of the positive benefits of reducing depressive symptoms for prognosis after orthopedic intervention, could yield both economic and patient quality of life gains that are likely to be particularly beneficial for women. In the longer term, focused inquiry in both preclinical and human studies into the co-occurrence and mechanistic underpinnings of depression and musculoskeletal disorders, including recovery from a primary orthopedic injury, will provide enhanced understanding of the biological drivers of this comorbidity, providing foundational information to design interventions that can lead to improved outcomes from both depression and musculoskeletal compromise with the elevated potential for particular benefit for women.

## Version 1 09/17/2024

Electronic publication
